# Effect of High Intensity Interval and Continuous Swimming Training on Body Mass Adiposity Level and Serum Parameters in High-Fat Diet Fed Rats

**DOI:** 10.1155/2016/2194120

**Published:** 2016-01-19

**Authors:** Guilherme L. da Rocha, Alex H. Crisp, Maria R. M. de Oliveira, Carlos A. da Silva, Jadson O. Silva, Ana C. G. O. Duarte, Marcela Sene-Fiorese, Rozangela Verlengia

**Affiliations:** ^1^Post-Graduate Program in Human Movement Sciences, Methodist University of Piracicaba (UNIMEP), Campus Taquaral, Rodovia do Açucar, km 156, Piracicaba, SP, Brazil; ^2^Post-Graduate Program in Food and Nutrition, Sao Paulo State University (UNESP), Araraquara, SP, Brazil; ^3^Department of Physical Education and Human Motricity, Federal University of Sao Carlos (UFscar), Sao Carlos, SP, Brazil; ^4^Institute of Physics of Sao Carlos, University of Sao Paulo (USP), Sao Carlos, SP, Brazil

## Abstract

This study aimed to investigate the effects of interval and continuous training on the body mass gain and adiposity levels of rats fed a high-fat diet. Forty-eight male Sprague-Dawley rats were randomly divided into two groups, standard diet and high-fat diet, and received their respective diets for a period of four weeks without exercise stimuli. After this period, the animals were randomly divided into six groups (*n* = 8): control standard diet (CS), control high-fat diet (CH), continuous training standard diet (CTS), continuous training high-fat diet (CTH), interval training standard diet (ITS), and interval training high-fat diet (ITH). The interval and continuous training consisted of a swimming exercise performed over eight weeks. CH rats had greater body mass gain, sum of adipose tissues mass, and lower serum high density lipoprotein values than CS. The trained groups showed lower values of feed intake, caloric intake, body mass gain, and adiposity levels compared with the CH group. No significant differences were observed between the trained groups (CTS versus ITS and CTH versus ITH) on body mass gains and adiposity levels. In conclusion, both training methodologies were shown to be effective in controlling body mass gain and adiposity levels in high-fat diet fed rats.

## 1. Introduction

Obesity is a chronic disease of multifactorial origin, dependent on the complex interaction between genetic, environmental, and epigenetic factors [1, 2]. Defined as the excessive accumulation of adipose tissue in the body [3, 4], physical inactivity and excessive high-energy food intake are considered the most important causes of obesity [2, 5, 6].

Obesity is associated with systemic low-grade inflammation and metabolic syndrome [7, 8]. The combination of these symptoms increases the risk of cardiovascular diseases, type 2 diabetes, and certain types of cancer, resulting in a reduction of life quality and expectancy [8–10].

Among nondrug treatment strategies for the control of energy balance and obesity, we highlight dietary interventions (reductions of food intake) and physical exercise [11, 12]. It is well established that continuous physical exercises performed at low/moderate intensities and high volume result in several adaptations on cardiorespiratory and muscular systems, favoring body fat oxidation and body mass loss [13–15].

On the other hand, evidence indicates that high-intensity and low-volume interval training induces similar or even superior muscular and physiologic adaptations compared to traditional continuous training in humans [16–18]. However, there is no consensus about the best exercise protocols for obesity treatment or control.

In the literature, few studies analyzed the effects of high-intensity and low-volume interval training in rats [19–22], and these studies confirmed a significant influence of interval training on muscular adaptations. Recently, Shen et al. [23] showed that high-intensity interval treadmill exercises resulted in lower values of fat mass and adipocytes sizes compared to moderate continuous exercise in high-fat diet fed Sprague-Dawley rats. However, the total distance covered was equalized between exercises protocols [23].

High-intensity interval training involves intermittent bouts of physical exercises (near maximal or supramaximal efforts) interspersed by periods of rest interval [24]. Therefore, the training volume (total time or distance covered) is considerably lower in high-intensity interval protocols when compared to traditional low/moderate intensity continuous training. The equalization of volume to compare the effects of interval and continuous training does not represent a practical implication of the reality of what is done in physical training protocols.

Understanding the effects of high-intensity/low-volume interval training and low-to-moderate intensity/high volume continuous training on body mass gain and adiposity levels will foment the elaboration of more efficient physical training interventions for the obesity control, since interval training is often suggested as a better time-efficiency protocol. Therefore, this study aimed to investigate the effects of interval and continuous swimming training on body mass gain and adiposity levels of rats fed with a high-fat diet. We hypothesized that the changes on body mass and adipose tissue would be similar between swimming training protocols treatments, despite the considerably low-volume on interval training protocol.

## 2. Material and Methods

### 2.1. Animals

Eight-week-old male Sprague-Dawley rats (*n* = 48 with mean body mass 347.0 ± 32.7 g) were obtained from the State University of Campinas (Universidade Estadual de Campinas (UNICAMP)). The animals were maintained on light/dark cycle of 12 h (light cycle between 6:00 and 18:00 hours) and under controlled temperature environment (21 ± 2°C) during this study. The experimental procedures were conducted in accordance to the 1996 Guide for the Care and Use of Laboratory Animals [25] and approved by local Ethics Committee for animal Research (protocol number 14/2013).

### 2.2. Experimental Design

The experimental design in the current study is commonly used to induce dietetic obesity in high-fat fed rats, since it promotes similar metabolic changes as observed in human obesity [2, 26].

Initially, during a four-week period, animals were randomly divided in two groups: standard diet group (SD, *n* = 24) and high-fat diet group (HD, *n* = 24). We housed one animal per cage with free access (*ad libitum*) to water and respective diet. This four-week period was used to induce changes in body mass by high-fat diet.

After this period, animals were randomly divided into six experimental groups: control standard diet (CS, *n* = 8), control high-fat diet (CH, *n* = 8), continuous training standard diet (CTS, *n* = 8), continuous training high-fat diet (CTH, *n* = 8), interval training standard diet (ITS, *n* = 8), and interval training high-fat diet (ITH, *n* = 8). The animals from trained groups (CTS, CTH, ITS, and ITH) performed their respective swimming training protocols for a period of eight weeks, while animals from control groups (CS and CH) were placed in shallow water (no exercise stimulus) for the same duration. Prior to the start of the physical training protocol, animals were adapted to liquid medium for a week, gradually increasing intensity and training volume.

At the end of study, animals were euthanized by decapitation 48 hours after the last exercise session and 8 hours without food. Blood samples were collected and serum was obtained for biochemical analysis. Additionally, samples of retroperitoneal, epididymal, and mesenteric white adipose tissues were removed and weighted.

### 2.3. Diet Composition

The standard diet (3.05 kcal/g-digestible energy) contained 64.4% carbohydrate (stanch 54.4% and sucrose 10.0%), 23.1% protein (casein), 4.8% fat (soy oil), 0.30% vitamins, and 7.7% mineral (Presence-Purina, Paulínia, São Paulo, Brazil). The high-fat diet (5.35 kcal/g-digestible energy) contained 26.88% carbohydrates (starch 19.40% and sucrose 7.48%), 15.17% protein (casein 14.95% and L-cystine 0.22%), 57.20% fat (soy oil 6.73% and lard 50.47%), and vitamins 0.75% (PRAGSOLUÇÕES-Bioscience, Jaú, São Paulo, Brazil). Nutritional information was provided by manufacturers.

### 2.4. Body Mass and Food Intake

Body mass and food intake (difference between the feed offered and the remaining feed) of each animal were measured daily throughout the experimental period by precision balance (Gehaka, model BG 2000, Brazil). Feed efficiency and energy efficiency were calculated using the following formulas:

(1) Feed Efficiency=body mass gain gtotal food intake g,Energy Efficiency=body mass gain gtotal caloric intake kcal.

### 2.5. Physical Training Protocols

Swimming training sessions were performed in an individual PVC chamber (60 cm high × 30 cm diameter) with water temperature controlled at 31 ± 1°C. The swimming training was conducted during eight weeks, with a weekly frequency of five days (Monday to Friday) during the morning (~8:00 and 11:00 AM).

The load intensity was attached to the animal's tail and was individually adjusted in each exercise session according to animal body mass. The progression of load intensity, volume of interval, and continuous training were adapted from protocols describe by Terada et al. [27] and Carnevali et al. [21]. A pilot study (*n* = 8) was previously conducted to test the applicability and the necessary adjustments to the training protocols proposed in this study. Regarding load intensity, a previous study [28] indicated that lactate threshold was achieved with loads between 5 and 6% of rats' body mass. Therefore, the continuous protocol used in this study was considered of low/moderate intensity (load between 0 and 3% of body mass) and interval protocol was considered as high intensity (load between 5 and 16% of body mass).  Table 1 shows swimming training protocols used during this study. After the exercise sessions, animals were dried and returned to the bioterium standard conditions.

### 2.6. Serum Biochemical Analysis

Serum concentrations of total cholesterol, HDL-cholesterol, triglycerides, glucose, and C-reactive protein (CRP) were analyzed using a commercial kit (Bioclin, Belo Horizonte, Minas Gerais, Brazil) according to the manufacturer's instructions. For this analysis, we used a semiautomated equipment (Bioclin 100, Belo Horizonte, Minas Gerais, Brazil).

### 2.7. Statistical Analysis

Data normality was assessed by the Shapiro-Wilk test. Independent *t*-test was used to compare the values between SD and HD groups. One way analysis of variance (ANOVA) followed by Bonferroni post hoc test was used for comparisons between groups at the end of study. Nonparametric data were analyzed by Kruskal-Wallis test (post hoc Dunn's test). Data are expressed as mean ± standard deviation. The significance level was set at *p* ≤ 0.05.

## 3. Results

### 3.1. Effects of Diet Type on Body Mass and Feed Intake

The body mass gain, feed intake, and feed and energetic efficiency during the first 4 weeks of our study are shown in Table 2. The high-fat diet resulted in greater final body mass and body mass gain values (HD > SD group). The total feed intake was higher in SD fed rats than in the HD fed rats. On the other hand, the total caloric intake and feed and energetic efficiency was significantly higher in HD fed rats.

At the end of study, the control high-fat fed rats showed greater final body mass, body mass gain, caloric intake, and feed efficiency than the control standard diet fed rats (CH > CS group; Table 3).

### 3.2. Effects of Diet and Physical Training Type on Body Mass and Feed Intake

The body mass gain, feed intake, and feed and energetic efficiency at the end of study are shown in Table 3. The trained groups (CTS, CTH, ITS, and ITH groups) showed lower final body mass, body mass gain, feed and caloric intake, and feed and energetic efficiency than the CH group.

For the rats fed with standard diet, the body mass gain and feed intake were lower for ITS group than in the CS group. On the other hand, caloric intake, feed, and energetic efficiency were not different between the trained groups (CTS and ITS) and the CS group.

When comparing the trained groups, CTS and ITS rats had higher feed intake than the CTH and ITH rats. In contrast, the values for final body mass, body mass gain, caloric intake, and feed and energetic efficiency were not significantly different among the treatment groups (ITS, ITH, CTS, and CTH; Table 3).

### 3.3. Effects of Diet Type on Adipose Tissue Mass

**Figure 1:**
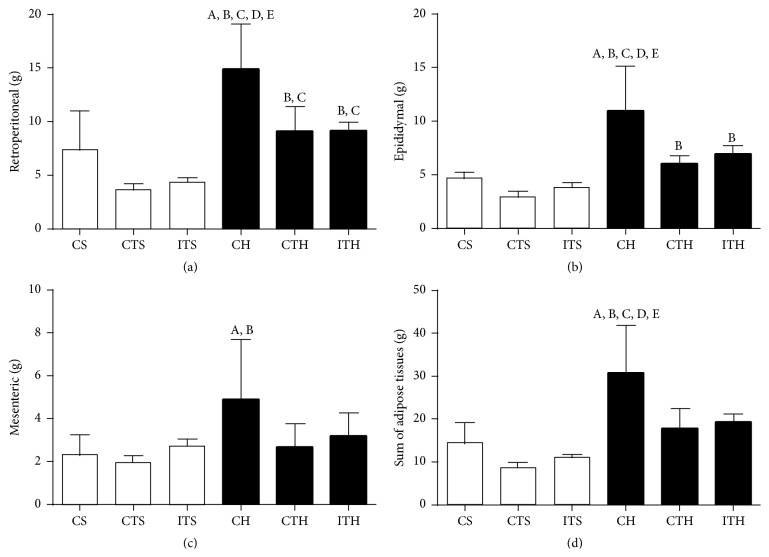
Absolute values of (a) retroperitoneal tissue, (b) epididymal tissue, (c) mesenteric tissue, and (d) sum of adipose tissues. CS: control standard diet; CTS: continuous training standard diet; ITS: interval training standard diet; CH: control high-fat diet; CTH: continuous training high-fat diet; ITH: interval training high-fat diet. ^A^ *p* < 0.05 compared to CS; ^B^ *p* < 0.05 compared to CTS; ^C^ *p* < 0.05 compared to ITS; ^D^ *p* < 0.05 compared to CTH; ^E^ *p* < 0.05 compared to ITH. Mesenteric values were compared by Kruskal-Wallis test. Values are means ± SD.

Figure 1 shows the absolute white fat mass: (a) retroperitoneal, (b) epididymal, (c) mesenteric adipose tissues, and (d) sum of adipose tissues. The CS group exhibited lower values for retroperitoneal, epididymal, and mesenteric adipose tissues and the sum of adipose tissue mass than the CH group.

### 3.4. Effects of Diet and Physical Training Type on Adipose Tissue Mass

The absolute retroperitoneal and epididymal adipose tissues mass and sum of adipose tissue mass were lower in the trained groups (CTS, CTH, ITS, and ITH groups) than the CH group. However, for mesenteric tissue, the difference was more evident in the CTS group than in the CH group, with no significant difference observed in the other trained groups (ITS, ITH, and CTH; Figure 1).

### 3.5. Effects of Diet Type on Biochemical Analyses

Table 4 shows the serum triglycerides, total cholesterol, HDL-cholesterol, glucose, and C-reactive protein levels. A significant difference was observed only for HDL-cholesterol, wherein the CH group showed lower values than the CP group.

### 3.6. Effects of Diet and Physical Training Type on Biochemical Analyses

The ITS group exhibited higher total cholesterol levels than the CTH group. Trained groups fed with high-fat diet (CTH and ITH groups) had significantly greater reductions in total cholesterol than the CH group. Additionally, the CTH group had higher HDL-cholesterol values than the ITH group.

Rats fed with standard diet (CS, CTS, and ITS groups) showed higher HDL-cholesterol levels than in the CH and ITH rats. No significant changes were observed for other triglycerides, glucose, and PCR findings among the groups (Table 4).

## 4. Discussion

This study aimed to investigate the effects of two different swimming training protocols (high intensity and low volume versus low/moderate intensity and high volume) on body mass gain, intra-abdominal adiposity levels, and serum biochemical parameters in high-fat diet fed rats. The main findings of study were as follows: (a) both physical training methods being effective in the control of body mass gain, (b) adiposity levels, and (c) serum total cholesterol concentration.

These results confirm our initial hypothesis and provide further evidence that physical exercise is an important nondrug strategy to control body mass gain and obesity. Furthermore, the shorter time of exercise period with high-intensity interval training is a time-efficient method to control changes associated with high-fat diet fed rats.

### 4.1. Effect of Diet

In our study, we used a saturated high-fat diet of animal origin to induce changes in body mass and obesity. In this sense, the total caloric intake values were higher for animals fed with high-fat diet during the first four weeks and at the end of the study. These data indicate that the high caloric density present in the high-fat diet (5.35 versus 3.05 kcal/g) was an important factor to promote significant changes in the Sprague-Dawley rats. Thus, animals from CH group showed greater values of final body mass, body mass gains, and adiposity levels when compared to CS group (Table 3 and Figure 1). Results corroborate with other studies that used similar diet [29–32], confirming the effectiveness of high-fat diet to promote dietary obesity in rats.

Regarding blood analysis, no statistical differences were observed for serum triglycerides, total cholesterol, glucose, and PCR values between CH and CS groups. However, CH group exhibited lower values for HDL-cholesterol compared to CS group (Table 4). Data from biochemical analysis is controversial in the literature, since some studies corroborate with our data [31, 33–36], also indicating no significant changes in triglycerides, total cholesterol, glucose concentration, and PCR, while, on the other hand, other studies [29, 32, 37] reported significant changes in these parameters using a diet similar to our study.

Thus, factors such as (a) the lineage of animals, (b) duration of the diet, and (c) fat concentration and type are variables that must be taken into consideration, which may have influenced the outcomes of our study. In the diet and experimental models adopted in this study, animals from CS and CH groups showed similar systemic metabolic adjustments (triglycerides, total cholesterol, glucose, and PCR), with significant changes only in HDL-cholesterol concentration, despite the significant increase in body mass gain and adiposity levels.

### 4.2. Effects of Exercise and Diet

CTH and ITH groups showed a reduction in feed intake and consequently on the caloric intake compared to the CH group (Table 3). In accordance with these data, other studies also showed that high-fat diet fed to rats reduces feed and caloric intake, when undergoing a period of physical training [33, 38]. This condition can be explained because physical exercise, a stress agent, may modulate the release of anorectic hormones and/or increase its receptors sensitivity, suppressing the appetite and controlling the energy balance during the physical training program [39, 40].

In this regard, CTH and ITH groups had lower values for final body mass, body mass gain, and adiposity levels (retroperitoneal and epididymal adipose tissues and the sum of adipose tissues) than the CH group (Table 2 and Figure 1). These data reinforce the importance of reducing caloric intake and increasing energy expenditure through interval and continuous training in order to control obesity in high-fat diet fed animals, since the energy balance (intake versus expenditure) is recognized as a crucial factor [11, 41, 42].

The current results are in contrast to those of Shen et al. [23] who reported significantly lower body mass gains, mesentery, retroperitoneal mass, and retroperitoneal adipocytes sizes in high-intensity treadmill interval training when compared to continuous training, in high-fat diet fed Sprague-Dawley rats. Additionally, in Shen et al.'s study [23], the caloric intake did not differ between trained and control groups, and the difference was attributed only to the physical training intervention. An important aspect that should be taken into account was that the interval and continuous training protocols were equalized for the total distance covered, and this provides a logical explanation for differences in outcome between protocols [23].

In our study, swimming protocols were conducted in an individual chambers tank to train the rats, and the discrepancy for intensity and volume between protocols was intentional. The duration of the exercise during interval training protocols lasted less than 5 minutes during the eight-week training period, while continuous training protocols ranged between 30 and 60 min. Thus, our proposed interval protocol can be considered as a very low-volume exercise. An interesting finding in our study was that, despite the exercise periods being much lower in high-intensity interval training, changes in body mass and adiposity level caused by high-fat diet were controlled in a similar magnitude as the traditional continuous training (low/moderate intensity and higher volume).

On the other hand, there was no influence of both physical training methodologies on mesenteric adipose tissue mass (Figure 1). The lipolytic activity is variable in different adipose tissues during physical exercise, which suggests that the visceral fat tissue had lowered its contribution as an energy source [43–45]. We could observe this in our study, since the mesenteric (more visceral) tissue was not significantly reduced in CTH and ITH groups when compared to CH (Figure 1). These data are consistent with other studies [46, 47], which used the continuous swimming training in high-fat diet fed rats and also did not find significant effects on mesenteric tissue mass.

Blood lipids disorders are critical risk factors for cardiovascular diseases, and physical exercise is recognized to modulate positive responses [12, 37, 46, 48]. In our study, there was a significant reduction in both trained groups (TCH and TIH) on total cholesterol concentrations in relation to the CH group (Table 4). On the other hand, only CTH showed higher HDL-cholesterol concentrations when compared to CH group. These data indicate better effectiveness of continued training on HDL-cholesterol levels of animals fed with a high-fat diet. However, the same response was not observed for trained animals fed with a standard diet (CTS versus ITS).

For the standard diet groups, no difference was observed for the caloric intake between groups (CS, CTS, and ITS) (Table 3). Thus, interval and continuous swimming training (CTS and ITS groups) did not affect significantly the final body mass, body mass gain, adiposity level (retroperitoneal, epididymal, and mesenteric tissue mass), and serum biochemical analyses, when compared to the CP group. Our data indicate that the proposed physical training protocols did not affect caloric intake in rats fed with standard diet and consequently on the investigated parameters.

These data differ from those obtained by Carnevali et al. [21] who investigated the effects of interval and continuous swimming training in standard diet fed Wistars rats. It was indicated that both trained groups (interval and continuous) had lower body mass gains and lower total cholesterol and triglyceride concentrations when compared to control rats. However, Carnevali et al. [21] did not report food and caloric intake between groups, a factor that may have influenced the final results of the study.

It is worth noting that, in the comparison between trained groups (CTS versus ITS and CTH versus ITH), no significant differences were evident. These data show that the magnitude of changes in the variables investigated in our study (body mass gain, adiposity levels, and biochemical variables) behaved similarly between interval and continuous training, indicating that both methods can be used to prevent obesity.

## 5. Conclusion

In conclusion, interval and continuous training were effective for controlling body mass gain, adiposity levels, and serum total cholesterol concentrations in high-fat diet fed Sprague-Dawley rats. Our data indicate that low-volume exercise (~5 min) can promote similar changes when compared to traditional continuous training (between 30 and 60 min), confirming the time efficiency when exercises are performed at high intensity. This strategy can be adapted and applied for obese individuals, which usually argue about the lack of time to engage in regular physical exercises programs.

## Figures and Tables

**Table 1:** Continuous and interval swimming training protocols.

Continuous swimming training				Interval swimming training				
Week	Set	Time	Load	Week	Set	Time	Rest	Load
1st	1	30 min	0%	1st	5	1 min	1 min	0–5%
2nd	1	40 min	0%	2nd	5	1 min	1 min	7%
3rd	1	30 min	1%	3rd	5	1 min	1 min	8%
4th	1	40 min	1%	4th	5	1 min	1 min	10%
5th	1	40 min	2%	5th	14	20 s	10 s	13%
6th	1	50 min	2%	6th	14	20 s	10 s	14%
7th	1	50 min	3%	7th	14	20 s	10 s	15%
8th	1	60 min	3%	8th	14	20 s	10 s	16%

**Table 2:** Mean values of body mass, feed intake, caloric intake, and feed and energetic efficiency during the first 4 weeks of our study (without exercise stimuli).

	SD (*n* = 24)	HD (*n* = 24)
Initial body mass (g)	347.8 ± 24.5	346.4 ± 39.1
Final body mass (g)	411.1 ± 23.0	449.6 ± 39.1^a^
Body mass gain (g)	63.2 ± 17.4	103.2 ± 27.0^a^
Feed intake (g)	706.3 ± 45.4	460.5 ± 47.1^a^
Caloric intake (kcal)	2154.2 ± 138.6	2463.8 ± 251.9^a^
Feed efficiency	0.090 ± 0.03	0.226 ± 0.06^a^
Energetic efficiency	0.029 ± 0.01	0.042 ± 0.01^a^

SD: standard diet; HD: high-fat diet. ^a^ *p* < 0.0001 compared to SD. Values are shown as mean ± SD.

**Table 3:** Mean values of body mass, feed intake, caloric intake, and feed and energetic efficiency during the 8-week training period.

	CS (*n* = 8)	CTS (*n* = 8)	ITS (*n* = 8)	CH (*n* = 8)	CTH (*n* = 8)	ITH (*n* = 8)
Initial body mass (g)	415.3 ± 25.9	408.6 ± 13.2	409.3 ± 13.2	458.7 ± 46.5	448.6 ± 39.5	441.4 ± 33.5
Final body mass (g)	472.0 ± 42.3	431.1 ± 33.1	425.3 ± 20.2	543.4 ± 52.1^a,b,c,d,e^	449.0 ± 22.9	450.8 ± 27.7
Body mass gain (g)	56.7 ± 29.9	22.5 ± 20.1	16.0 ± 18.9^a^	84.8 ± 19.0^a,b,c,d,e^	21.17 ± 29.9	10.6 ± 15.7
Feed intake (g)	1258.6 ± 70.4	1188.2 ± 65.6	1162.4 ± 54.8^a^	793.9 ± 36.5^a,b,c,d,e^	683.3 ± 41.9^a,b,c^	639.7 ± 56.6^a,b,c^
Caloric intake (kcal)	3838.8 ± 214.7	3623.9 ± 199.9	3545.3 ± 167.2	4247.3 ± 195.3^a,b,c,d,e^	3655.7 ± 224.3	3422.4 ± 302.6
Feed efficiency	0.045 ± 0.024	0.018 ± 0.017	0.014 ± 0.016	0.108 ± 0.028^a,b,c,d,e^	0.029 ± 0.04	0.016 ± 0.023
Energetic efficiency	0.015 ± 0.008	0.006 ± 0.005	0.005 ± 0.005	0.020 ± 0.005^b,c,d,e^	0.005 ± 0.008	0.003 ± 0.004

CS: control standard diet; CTS: continuous training standard diet; ITS: interval training standard diet; CH: control high-fat diet; CTH: continuous training high-fat diet; ITH: interval training high-fat diet. ^a^ *p* < 0.05 when compared to CS; ^b^ *p* < 0.05 when compared to CTS; ^c^ *p* < 0.05 when compared to ITS; ^d^ *p* < 0.05 when compared to CTH. ^e^ *p* < 0.05 when compared to ITH. Values are shown as mean ± SD.

**Table 4:** Serum lipid profile, glucose, and C-reactive protein.

Groups	TG (mg/dL)	TC (mg/dL)	HDL-C (mg/dL)	Glucose (mg/dL)	PCR (mg/dL)
CS	119.9 ± 19.6	84.6 ± 6.8	48.9 ± 6.4	105.0 ± 12.8	0.72 ± 0.1
CTS	110.6 ± 27.7	78.3 ± 10.5	56.8 ± 5.6	101.7 ± 17.8	0.70 ± 0.1
ITS	119.8 ± 18.0	85.9 ± 9.5	50.4 ± 7.6	101.6 ± 14.2	0.70 ± 0.3
CH	119.3 ± 43.8	90.0 ± 14.0^d,e^	39.2 ± 5.4^a,b,c^	110.0 ± 11.6	0.70 ± 0.2
CTH	95.9 ± 18.4	70.3 ± 6.8^c^	47.3 ± 9.7	100.9 ± 9.2	0.70 ± 0.2
ITH	105.7 ± 30.9	73.1 ± 6.0	35.9 ± 3.8^a,b,c,d^	102.0 ± 5.1	0.75 ± 0.2

TG: triglycerides; TC: total cholesterol; HDL-C: HDL-cholesterol, PCR: protein C-reactive. ^a^ *p* < 0.05 compared to CS; ^b^ *p* < 0.05 compared to CTS; ^c^ *p* < 0.05 compared to ITS; ^d^ *p* < 0.05 compared to CTH. ^e^ *p* < 0.05 compared to ITH. Values are means ± SD.
